# The Role of Microbiome in Insomnia, Circadian Disturbance and Depression

**DOI:** 10.3389/fpsyt.2018.00669

**Published:** 2018-12-05

**Authors:** Yuanyuan Li, Yanli Hao, Fang Fan, Bin Zhang

**Affiliations:** ^1^Key Laboratory of Mental Health and Cognitive Science of Guangdong Province, and School of Psychology, Center for Studies of Psychological Application, South China Normal University, Guangdong, China; ^2^Department of Anatomy, Guangzhou Medical University, Guangdong, China; ^3^Department of Psychiatry, Nanfang Hospital, Southern Medical University, Guangdong, China

**Keywords:** gut microbiome, microbiome-gut-brain axis, insomnia, depression, mental health, circadian rhythm, metabolic disease, inflammation

## Abstract

Good sleep and mood are important for health and for keeping active. Numerous studies have suggested that the incidence of insomnia and depressive disorder are linked to biological rhythms, immune function, and nutrient metabolism, but the exact mechanism is not yet clear. There is considerable evidence showing that the gut microbiome not only affects the digestive, metabolic, and immune functions of the host but also regulates host sleep and mental states through the microbiome-gut-brain axis. Preliminary evidence indicates that microorganisms and circadian genes can interact with each other. The characteristics of the gastrointestinal microbiome and metabolism are related to the host's sleep and circadian rhythm. Moreover, emotion and physiological stress can also affect the composition of the gut microorganisms. The gut microbiome and inflammation may be linked to sleep loss, circadian misalignment, affective disorders, and metabolic disease. In this review article, we discuss various functions of the gut microbiome and how its activities interact with the circadian rhythms and emotions of the host. Exploring the effects of the gut microbiome on insomnia and depression will help further our understanding of the pathogenesis of mental disorders. It is therefore important to regulate and maintain a normal gastrointestinal micro-ecological environment in patients when treating mental disorders.

## Introduction

In recent years, the role of the brain-gut axis in neuropsychiatric disorders has received increasing attention. Epidemiological studies have shown that functional dyspepsia (FD) and irritable bowel syndrome (IBS) are closely related to psychosocial factors. Over 50% of patients with IBS have comorbid depression, anxiety or sleep problems (1–3). Psychological counseling or antidepressant therapy is effective for some patients with IBS (4, 5). Depression and generalized anxiety disorder (GAD) are often associated with gastrointestinal disturbances (6, 7). An increasing number of studies suggest that the intestinal microbiota can regulate sleep and mental state through the brain-gut axis. Though gaining increasing popularity, research on how the intestinal microbiota contribute to mental disorders is still in its infancy. Elucidating the mechanisms that underlie the interactions of the intestinal microbiota and the nervous system helps us understand sleep and affective disorders and find optimized ways to treat them (8). This review summarizes the role of the microbiome in sleep and mental disease, focusing on the interaction of the microbiome with circadian rhythms and sleep problems. The purpose of this review is to provide a basis for understanding the effects of intestinal microbes on human mental and sleep disorders.

## The Gut Microbiome and the Gut-Brain Axis

The term “gut microbiome” is generally used to refer to the microorganisms that inhabit the gastrointestinal tract, including their genetic material (9). Approximately 1,000 types of microbiota are present in the adult intestinal tract. The most abundant species belong to the phyla Firmicutes and Bacteroidetes. Other abundant species belong to the Proteobacteria, Actinobacteria, Fusobacteria, Verrucomicrobia, and Cyanobacteria (10). The composition of the intestinal microbiota shows a dynamic equilibrium in a healthy condition (11–13). The delicate balance of the microbiota is very important for health because dysbacteriosis increases the host's susceptibility to disease.

**Figure 1:**
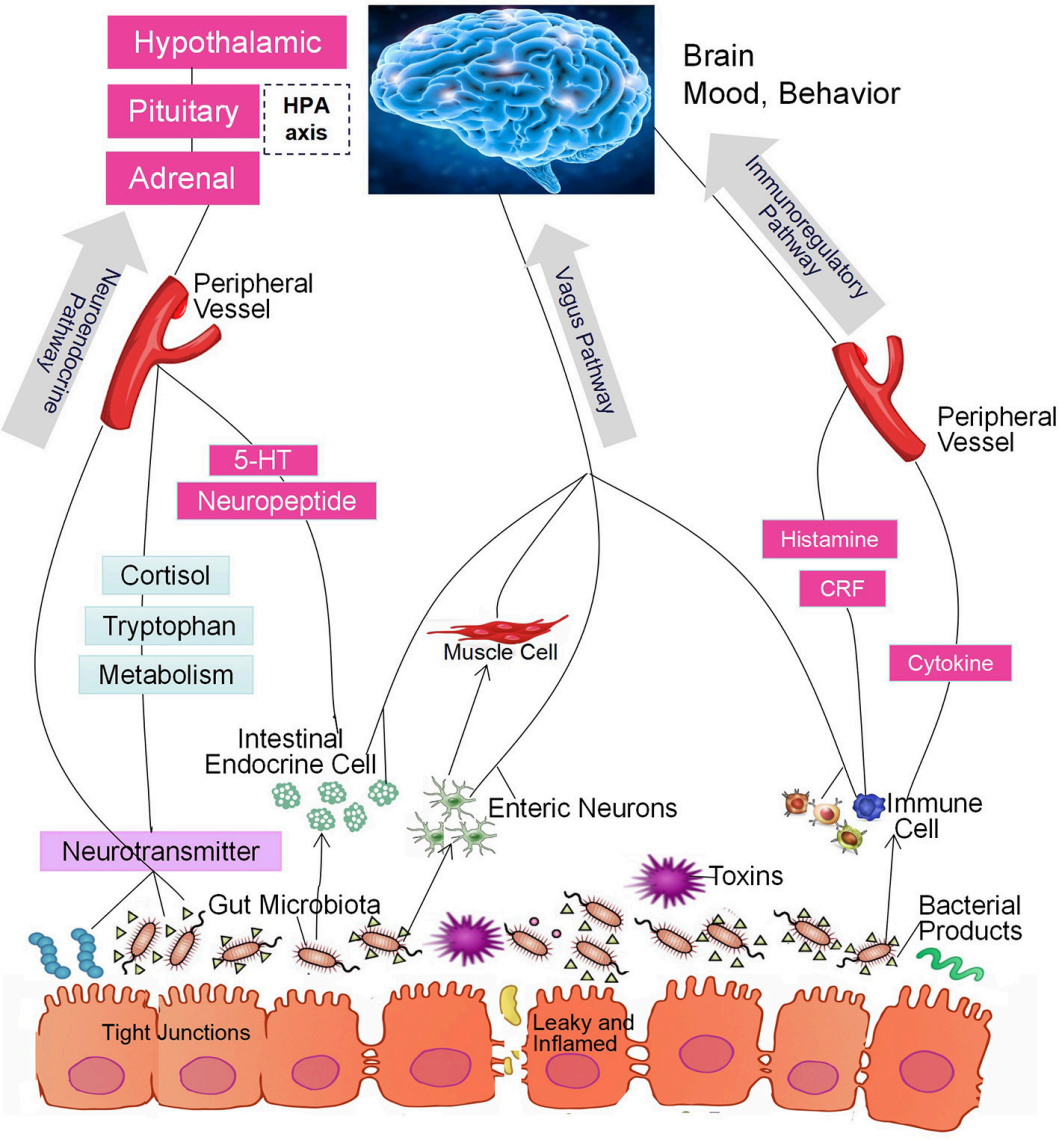
The intestinal microbiota regulate brain function through the microbiome-gut-brain axis of the immunoregulatory, neuroendocrine, and vagus pathways.

In the past decade, a large number of studies have identified a microbiome-gut-brain (MGB) axis (14, 15). Within this axis, the microbiota in the gut affect brain function through 3 pathways that produce a bidirectional flow of information (16–20). The first of these is the immunoregulatory pathway, in which the microbiota interact with immune cells in such a way as to affect the levels of cytokines, cytokinetic reaction factor, and prostaglandin E2 (21). As a result, brain function is affected. The second is the neuroendocrine pathway. There are more than 20 types of enteroendocrine cells in the intestine, which constitutes the largest endocrine organ in the human body (22). The gut microbiome may affect the hypothalamic-pituitary-adrenal (HPA) axis and the central nervous system (CNS) by regulating the secretion of neurotransmitters such as cortisol, tryptophan, and serotonin (5-HT). The third is the vagus nerve pathway, in which the enteric nervous system plays an important role (23). Anatomical evidence indicates that the sensory neurons of the intestinal myenteric plexus are exposed to the gut microbiota; these neurons form synaptic contacts with motor neurons in the intestine that are involved in the regulation of intestinal motility and gut hormone secretion. The intestinal nervous system also forms synaptic connections with the vagus nerve, which connects the intestine to the brain (23) and constitutes an information transmission pathway that could be described as the gut microbiota-enteric nervous system (ENS)-vagus-brain pathway. Furthermore, neurotoxic metabolites such as D-lactic acid and ammonia produced by the gut microbiota may pass through the vagus nerve into the CNS, thereby affecting brain function, stress responses, and sleep structure (24, 25). Similarly, the CNS can also regulate the composition of the intestinal microbiota through these three pathways. For example, the HPA axis regulates intestinal peristalsis and controls epithelial cell functions, thereby affecting the environment of the intestinal microbiota, including the intestinal permeability, and further changing the composition of the gut microbiota (14, 15, 26, 27). The 3 pathways are summarized in Figure 1. It should be noted, however, that most studies did not test the function of each pathway. Thus, although the intestinal microbiota may affect brain function through a specific pathway, we cannot exclude the possibility that other pathways may also be involved (28, 29).

## The Gut Microbiota, Clock Genes and Sleep

The intestinal microbiota exhibit circadian rhythms in both population structure and functional activity. Evidence suggests that Clostridiales, Lactobacillales, and Bacteroidales, which account for ~60% of the microbiota, show significant diurnal fluctuations that result in time-of-day-specific taxonomic configurations (30). Liang et al. (31) found that the two primary components of the mammalian intestinal microbiota, Bacteroidetes, and Firmicutes, showed cyclical changes in abundance from day to night that are related not only to rhythmic food intake and dietary structure (30, 32, 33) but also to the biological clock (34) and gender (31) of the host.

Recent studies showed that circadian clock misalignment, sleep deprivation, and shift experience changes circadian clock gene expression and microbial community structure (35–38). Interfering with the sleep patterns of mice can also change the structure and diversity of the intestinal microbiota (32). These findings suggest that circadian genes might affect the intestinal microbiota.

The intestinal microbial community exhibits rhythmic fluctuations in its structure and function. As a result, the intestinal epithelium is exposed to different bacterial species and their metabolites throughout the day. In turn, the circadian rhythm of the microbiota drives the transcription of host circadian clock genes and affects epigenetic modifications and oscillations in metabolite levels (32). Therefore, a specific link between the intestinal microbiota and the host circadian clock must exist.

Depression and sleep are both closely related to circadian activity. For example, depressed patients often experience milder symptoms at night and more severe symptoms in the morning. The incidence of depressive symptoms among shift workers is significantly higher than that among normal workers (39). In addition, the clinical manifestations of seasonal affective disorder are depressive symptoms related to specific seasons. Furthermore, the onset of this disorder is also related to biological rhythms such as day length and the intensity of ambient light (40, 41). This evidence indicates a close relationship between depression and biological rhythms. Depressive episodes are also often associated with sleep disorders. For example, depressive patients might have decreased sleep time, increased rapid eye movement (REM) sleep or shorter REM latency (42). However, the specific mechanisms of comorbidity of sleep disorders and depression are still unknown.

Microbes and circadian genes are inextricably intertwined (32, 43). Firstly, the disruption of the host circadian rhythm alters the gut microbiome equilibrium. These changes are similar to those that occur with actual shift experience (44, 45). In addition, the body's biological clock works in synergy with the microbial clock. Thaiss et al. found that the population and functions of the intestinal microbiota lost circadian rhythm in *Per1/2*^−/−^ mice (30). In the study of Liang et al. (31), the fecal microbiota of clock gene *Bmall* knockout mice showed significant changes in the rhythmicity of total load and taxonomic abundance. These results suggest that some host clock genes such as *Bmall, Per1*, and *Per2* are closely related to changes in intestinal microbiological rhythms. In addition, the interaction of the microbiota and the circadian clock may have a profound effect on host metabolism. Recent studies have shown that host microbiota increase host metabolism by affecting the circadian transcription factor NFIL3 (43). This results may help explain why the prevalence of metabolic syndrome is higher in shift workers and in people who often experience jet lag.

In addition, the microbiome is able to mediate host clock gene expression in the suprachiasmatic nucleus (SCN). Germ-free (GF) mice are an animal model in which microbiota are deficient from birth. Compared with conventional experimental animals, GF animals have a clear microbiological background, and distinct features in terms of anatomy, morphology, metabolic physiology, the immune system, and biological rhythms. Therefore, GF mice provide a good model for studying the effects of the gastrointestinal microbiome on host brain activity. Leone et al. (34) demonstrated that oscillations in the expression of circadian clock genes in the SCN were reduced in GF mice. Additionally, clock genes mutations promote intestinal dysbiosis. In mice, Voigt et al. (46) found that core clock genes mutation caused gut microbiome dysbiosis and that this trend was exacerbated by dietary intestinal stimuli. This result shows that the gut microbiome plays a crucial role in maintaining the normal expression of host clock genes. The above findings demonstrate that gut microorganisms are able to mediate the host clock gene regulatory network, resulting in various metabolic pathological alterations, and vice versa.

Many studies have shown that in depressive patients, polymorphisms of clock genes are affected by hyperactivation of the HPA axis, which can initiate sleep dysfunction and further plays an important role in the development of depression (47–49). There is no doubt that the circadian clock genes are closely related to the development of sleep disorders such as insomnia. Preclinical studies have also suggested that the clock genes are associated with sleep rhythm disturbances in patients with affective disorders. In patients with depression, the rhythm of clock genes expression is disrupted, resulting in clinical symptoms similar to those associated with jet lag (39). The circadian clock genes are also associated with dopamine, an important molecule related to depression. For example, the lack of a functional CLOCK protein enhanced dopamine release in the striatum (50); CLOCK knockout mice displayed increased risk of depression together with significant changes in dopamine-related gene expression in the ventral tegmental area (51). Moreover, clock genes expression in some brain regions is susceptible to modulation via epigenetic regulation and environmental factors. For example, in depressive patients, the amplitudes of oscillation in the expression levels of core clock genes (*Bmall* and *Per1-3*) were significantly decreased in the dorsolateral prefrontal lobe, amygdala, cerebellum, nucleus accumbens, anterior cingulate cortex, and hippocampus (52).

## Gut Microbiota, Stress and Anxiety

Stress is an important cause of insomnia and depression. Patients with sleep disorders or depression often have anxiety symptoms as well. When facing stress, these patients tend to be anxious at first; depression and sleep problems then often worsen. Recently, researchers found that intestinal dysbacteriosis is related to stress and anxiety (8, 28, 52–56). Shift workers, for example, usually experience short sleep times and disturbed circadian rhythms. These issues may cause a series of physiological stress reactions that alter the gut microbiota composition. The altered microbiota may affect the function of both the nervous and immune systems, thereby reducing an individual's ability to cope with psychological and physical stress and making him or her more vulnerable to stressful life events (44). However, some probiotics, such as *Lactobacillus, Bifidobacterium*, and *Enterococcus*, may cause changes in emotional and cognitive indicators by acting on the ENS and the immune system, thereby improving stress response and exerting an anti-anxiety effect (57).

In animal studies, the gut microbiome composition was found to be altered when neonatal rats were isolated from their mothers (58). Acute and chronic stress increased gut mucosal permeability and bacterial translocation in clinical and laboratory environments (53, 55). Under chronic stress conditions, mice showed anxiety-like behavior and displayed changes in the quantity and distribution of their intestinal microorganisms (56). These maladaptive responses to stress in mice were partially corrected by reconstitution of the gut microbiota with fecal bacteria obtained from control mice. Notably, as in the case of GF mice, different studies have reported different anxiety-like behaviors of the animals in response to stress. For example, Crumeyrolle-Arias et al. (59) reported that the absence of gut microbiota exacerbates neuroendocrine and anxiety-like behavioral responses to acute stress, while Clarke et al. (60) reported anxiolytic behavior of GF mice under other anxiety test conditions. Such discrepancies in complex anxiety behavior could result from methodological differences and different genetic backgrounds of the tested animals (61, 62).

Other studies have shown that corticosterone and melatonin are involved in the interaction of intestinal microbiota with stress and anxiety conditions (63–65). Imbalances of glucose and lipids often occur in shift workers (66). At the same time, their cortical rhythms were lost, suggesting that loss of diurnal rhythmicity may contribute to the physiological stress responses of the human body, leading to metabolic dysfunction, and corticosterone plays an important role in this process (66). Probiotic intervention decreased stress-induced corticosterone secretion and had anxiolytic and antidepressant-like effects in mice exposed to chronic stress (28, 58). Recent studies reported that clock genes regulate the rhythmic production of cortisol independent of the HPA axis in intestinal epithelial cells. Microbiota compositional dysbiosis was shown not only to disrupt circadian gene expression but also to affect the phasic production of ileal corticosterone, leading to sustained high levels of cortisol hormones (67). It is well known that cortisol is closely related to stress and anxiety; thus, corticosterone may be a bridge that connects the intestinal microbiome to stress and anxiety. Weaning exerts stress on growing mice. The weaning mice lost weight, had disrupted gastrointestinal microbiomes, and exhibited anxiety-like behavior, but melatonin supplementation was able to reverse these effects. The linear discriminant analysis effect size (LEfSe) revealed that melatonin supplementation significantly increased the richness of the microbial community and the relative abundance of *Lactobacillus intestinalis, Lactobacillus johnsonii*, and *Lactobacillus reuteri*, while it reduced the relative abundance of Prevotellaceae (67). Therefore, melatonin might alleviate weaning stress in mice by regulating the intestinal microbiota (68–72).

## Gut Microbiota and Comorbity of Insomnia and Depression

Depression and insomnia often occur together. Insomnia and poor sleep quality are the most common complaints of patients with depression (73). Additionally, insomnia have been shown to increase the severity of depression (74). In the general population, nearly 20% of insomnia patients also experience depressive symptoms (75). Although several hypotheses regarding the relationship between depression and insomnia have been proposed, including neurotransmitter depletion, HPA axis dysregulation, neural immune activation, and neurotrophin dysregulation, no single hypothesis is able to explain the complex mechanisms that have been observed in the field of neuropsychiatric research.

In recent years, many studies have suggested that the MGB axis may provide the basis for a hypothesis that explains the comorbidity of depression and insomnia. Microbial metabolism produces a variety of neurotransmitters, cytokines, and metabolites such as 5-HT, dopamine (DA), GABA, short-chain fatty acids (SCFAs), melatonin, and other compounds. Those metabolites not only act directly on the ENS and the vagus nerve but also affect the activity of the CNS by regulating enteroendocrine cells in autocrine or paracrine fashion (76). For example, 90% of the serotonin (5-HT) in the human body is derived from chromaffin cells in the gastroenteric tract, and some gut bacteria such as spore-forming bacteria are able to modulate the synthesis and secretion of 5-HT by acting on chromaffin cells (77). In addition, *Escherichia coli* and *Enterococcus*, which are common in the intestinal tract, also produce small amounts of 5-HT (78). It is well known that 5-HT is related both to the occurrence of REM sleep and to the development of depression (79).

*Lactobacillus* and *Bifidobacterium* can secrete GABA (80), and abnormal expression of GABA mRNA is often observed in patients with depression and insomnia. In animal experiments, mice fed *Lactobacillus rhamnosus* (JB-1) showed reduced anxiety- and depression-related behavior and altered cerebral expression of both GABA type A and GABA type B receptors compared with control mice (53). These changes were similar to benzodiazepine effects (81). However, the neurochemical and behavioral effects were not found in vagotomized animals, suggesting that the vagus nerve is a major communication pathway between gut bacteria and the brain (49). Screening for this type of enteric nervous system activity could possibly lead to the development of potential treatments for depression and insomnia.

Studies have confirmed that changes in the composition of the intestinal microbiome caused by antibiotics, probiotics (live bacteria), and special culture techniques appear to involve host anxiety or depression-like behavioral alterations (28, 82–84). These behaviors could be transferred from human hosts to mice through fecal microbiological transplantation, which can induce depression-like behavior in mice. For example, gavage of GF mice with feces from depressive patients was shown to lead to the spontaneous development of depression-like behavior in mice, and an animal model of humanized depression was thereby established. In addition, the characteristics of the gastrointestinal microbiome in the model mice were highly similar to those of patients with depression (85). These findings further confirm the association between depression and gut microbes and suggest that depression-like behavior can be transmitted between hosts though intestinal microbiota transplantation. Moreover, mice that received microbiota transplantation from depressive patients also showed abnormalities in tryptophan metabolism pathways (52). The metabolism of tryptophan is regulated by the MGB axis (27), and by-products produced by intestinal microbes during the breakdown of tryptophan can be transmitted to brain regions and influence the CNS (86). Furthermore, one research study also reported that antibiotics transiently decreased anxious behavior and altered brain BDNF expression in mice when given orally but not when given intraperitoneally, suggesting that the alteration in gut microorganisms and not the antibiotic itself was responsible for the effects (87).

Emotional stress and alteration of the host's circadian rhythms can, in turn, also trigger a change in the composition of the intestinal microbiota. Hyperactivity of the HPA axis caused by psychological or physiological stress (such as that produced by shift work and insomnia) can damage the gut-microbiota equilibrium by increasing gut mucosal permeability and activating intestinal immunity. Corticotropin-releasing hormone (CRH) acts on the intestinal plexus in a mast cell-dependent fashion, reduces the expression of claudins, and causes intestinal barrier dysfunction (88). When the intestinal permeability changes, lipopolysaccharide (LPS) is recognized by toll-like receptors (TLRs) on the surfaces of immune cells, causing the secretion of pro-inflammatory factors, which in turn causes an inflammatory response (39). Inflammation and infections by pathogens are the pathological basis for a variety of mental illnesses that can lead to anxiety, depression or sleep disorders. Zheng et al. (85) found that depressed patients had increased relative abundance of Actinobacteria and Firmicutes but decreased abundance of Bacteroidetes. Jiang et al. (89) found that, compared with controls, patients with depression showed significantly increased Shannon index scores for intestinal microbiota. Although the abundances of Bacteroidetes, Proteobacteria, and Actinobacteria were significantly increased, the abundance of Firmicutes was significantly decreased. Additionally, the level of *Faecalibacterium* was negatively correlated with the degree of depression in patients. These findings suggest that depression might damage the gut-microbiota equilibrium, reducing the anaerobic population and increasing the number of aerobic inhabitants. Another study also found a decrease in the abundance of intestinal *Bifidobacteria* and *Lactobacilli* in patients with depression (90). Furthermore, Voigt et al. (91) reported that circadian disorganization alters intestinal microbiota (91). Although the results of these studies are inconsistent, the inconsistency might be related to sample size, ethnic origin, dietary habits, and/or whether or not the subjects were taking antidepressants. Clinical samples from different sources have consistently suggested that depression appears to involve gut microbiome changes.

In summary, there is a bidirectional connection between the gut microbiome and sleep and depression. Inflammation and endocrine hormones play important roles in this process. First, chronic disturbances of host circadian rhythms, sleep loss, and depression have an impact in the metabolism of indigenous gut bacteria and trigger changes in their composition, typically reducing the total number of organisms of the Lactobacillaceae family but increasing the populations of *Bacteroides multiforme*, Enterococci, the Lachnospiraceae, and the Ruminococcaceae, which causes microbial dysbiosis (30, 34, 92). Second, gut bacteria can modulate the tight junctions between epithelial cells that reduce intestinal permeability and protect the intestinal barrier (53). Breakdown of the epithelial barrier can cause bacteria and their harmful metabolites to enter the mesenteric lymph tissue, stimulate inflammatory immune reactions and excite the vagus and spinal afferent nerves (93). One possible mechanism for this process may be the inflammatory reactions induced by gut microbiome dysbiosis, which result in abnormal immune reactions and further influence the CNS, causing or aggravating insomnia and depression (94–98) and driving metabolic disease.

Moreover, the intestinal microbiota interact with the clock genes in many ways. However, further study is required to determine how the rhythmic activity of the intestinal microbiota participates in the circadian rhythm control network of the intestine or the whole body. Additional research is also required to determine how rhythmic oscillations and functional activities are coordinated between the host and microbes.

## Sleep Loss, Circadian Misalignment, Affective Disorders and Metabolic Disease-Gut Microbiota: Is Inflammation the Link?

A growing body of evidence shows that circadian rhythm disorders, insomnia, and affective disorders are linked to a range of health problems, including obesity, diabetes, metabolic syndrome, and inflammatory diseases. Numerous epidemiological studies have indicated that shift workers are at increased risk of obesity (99–102), type II diabetes (103–106), and metabolic disease (107–110). The interaction between environmental clock disruption and chronic disruption of normal sleep and diet patterns was shown to cause obesity and metabolic disease in shift workers.

Many animal studies demonstrate that adverse metabolic effects occur when the balance is tipped toward increased numbers of pro-inflammatory gut bacteria. This imbalance is thought to promote increased gut permeability and the subsequent translocation of bacterial components across the intestinal epithelium. The consequence of this translocation process is exposure of metabolically active tissues (adipose tissue, the liver, muscle, and the pancreas) to low-level chronic inflammation, which can result in disturbed metabolic signaling, including insulin-mediated glucose uptake. As mentioned above, depression appears to impact the composition of the gut microbiome. McCaffery et al. (111) also found that depression could increase the risk of metabolic syndrome when they investigated the relationship between depression and metabolic risk factors in 173 pairs of twins. Perry et al. (112) found that nutrient-gut microbial interactions activated parasympathetic nerves, thus increasing acetate production in the body, which is accompanied by obesity and its sequelae. Therefore, the discovery of specific disease-associated microbiota and how the regulation of the intestinal microbial composition relates to metabolism may be a new scientific frontier. These discoveries may help establish methods for treating mental disorders and improving the quality of life of people with mental disorders.

Circadian rhythm disorders appear to damage the gut-microbiota equilibrium, and this type of damage is associated with the occurrence of metabolic syndrome. However, the damage can be ameliorated by antibiotic intervention or dietary adjustments. Summa et al. (113) found intestinal barrier dysfunction and disrupted gastrointestinal microbiomes in mice with disturbed circadian clocks; the disturbance was also accompanied by aggravated endotoxemia and fatty liver disease. However, the exact mechanisms that lead to these changes are unclear. Thaiss et al. (30) fed a high-fat diet to mice with disturbed circadian rhythms and to control mice. Compared to mice that maintained normal circadian rhythms, jet-lagged mice exhibited enhanced weight gain and exacerbated glucose intolerance. Treatment with broad-spectrum antibiotics (vancomycin, ampicillin, kanamycin, and metronidazole) reduced the weight gain and changed the glucose intolerance of jet-lagged mice.

Increase in permeability of the epithelial barrier (primarily caused by an increase in lipopolysaccharide (LPS) in the body and subsequent bacterial translocation) is an important cause of the characteristic feature of metabolic dysfunction—chronic low-level inflammation *in vivo*. Reduced sleep time can also cause alterations in gut microorganisms and increased permeability of epithelial barriers. For example, Poroyko et al. (92) found that chronic sleep fragmentation (SF) can induce increased food intake and reversible changes in the composition of the gut microbiome in mice; animals subjected to these conditions showed preferential growth of Lachnospiraceae and Ruminococcaceae and reduced numbers of Lactobacillaceae. These changes, in turn, led to systemic and visceral white adipose tissue inflammation and changes in insulin sensitivity, possibly due to disruption of the colonic epithelial barrier. In addition, GF mice that received fecal transplants from SF mice had increased inflammatory reactions and impaired metabolism, whereas administration of probiotics such as *Lactobacillus, Bifidobacterium*, and *Enterococcus* can reduce levels of inflammatory cytokines and combat the damaging inflammation caused by TLRs (88). These results demonstrate that SF-associated metabolic disruption is transmissible through microbiota and that probiotic intervention helps reduce the inflammatory response that is caused by metabolic disturbance.

Experimental results obtained in animals have prompted researchers to investigate whether microbiota are involved in metabolic imbalances associated with altered circadian rhythms in humans. However, related research on human subjects is still rare. One study (30) found that microbiota samples obtained from subjects experiencing jet lag showed a higher relative representation of Firmicutes and that this was reversed upon recovery from jet lag. Interestingly, Firmicutes have been associated with a higher propensity for obesity and metabolic disease in multiple human studies. Furthermore, when intestinal microbiota samples from individuals who had been experiencing jet lag for 24 h were transplanted into GF mice, the mice began to show weight gain and decreased glucose tolerance. However, the experimental design of this study did not control for many of the possible confounding factors, and the sample size was small (114, 115). Moreover, some studies of chronic fatigue syndrome (CFS) found that in addition to long-term unexplained fatigue, CFS patients also had poor sleep quality, daytime symptoms, and emotional and cognitive problems (116). Compared with healthy people, CFS patients had significantly reduced *Escherichia coli* and *Bifidobacterium* populations and significantly increased *Enterococcus* and *Candida albicans* populations during the acute phase of the disease (117). Yet, studies have shown that poor mood in CFS patients is also associated with higher levels of *Lactobacillus*. When patients were treated with antibiotics to relieve the imbalance in the intestinal microbiota, actigraphy records revealed that sleep efficiency, sleep time, and mood were improved (118). Although the sample size of the study was small and there were many confounding factors, these results suggest that disruption of the gastrointestinal microbiome might cause or prolong sleep problems.

Together, these preliminary data suggest that circadian misalignment and sleep loss in humans are associated with dysbiosis and that the resulting microbiota configurations may contribute to the occurrence of metabolic imbalances. The reduction of an individual's sleep time or the disruptions of host circadian rhythms will lead to a physiological stress response and change the host's normal intestinal microbiota. Furthermore, these changes will cause host inflammatory reactions, metabolic disorders, and impaired immune function. This process will also change the metabolism of neurotransmitters and cause nervous system dysfunction. The individual will then experience sleep problems or psychiatric symptoms, which ultimately initiate a vicious cycle.

## Limitations of Current Research

At present, research on the effects of the intestinal microbiota on the pathogenesis of some psychiatric disorders, such as depression and insomnia, is incomplete. For example, it remains unknown how the central biological clock network system interacts with the peripheral biological clock network system, how various biological clock signals are concentrated and transmitted in major peripheral organs, how these signals affect a series of downstream physiological reactions and ultimately result in microbial dysbiosis, and which subtypes of sleep disorders are affected by intestinal dysbacteriosis. To what extent mistimed sleep and affective disorders affect the molecular regulators of circadian rhythmicity and the intestinal microbiota also remains to be established. Furthermore, it remains unclear whether microorganisms respond directly to the body's circadian rhythm or whether they can change the rhythm of the host. It also remains unknown whether microbiological rhythms have substantial significance. In addition, many of the existing studies mainly focus on animal experiments and cross-sectional observations and examine only a single disease. Therefore, these studies are relatively isolated and do not constitute systematic investigations. Most are based on randomized sample collections and do not control for host aging, lifestyle, diet, concomitant disease (119, 120), antibiotic exposure, pet ownership (121) or other factors. There is no accurate test for sleep quality, and further verification by randomized controlled experiments is still required. The studies that have been conducted to date have discovered only indirect relationships between circadian misalignment, lack of sleep, affective disorders, and a disrupted gastrointestinal microbiome. Few studies have investigated how interactions between the intestinal microbiome and psychological and social environmental factors affect insomnia or the various pathological stages of insomnia. Finally, the mining and utilization of metagenomic data remain limited to the superficial level. The collation and analysis of genetic and epigenetic data from massive samples must be performed to provide personalized predictions for patients.

## Implications and Future Directions

In the past 20 years, rodent and human sleep studies investigating the health consequences associated with sleep loss and circadian misalignment have increased. With the increasing accessibility of high-throughput sequencing technology for quantifying microbiota, a growing body of research supports the idea that there is a bidirectional system in which good, adequate sleep, healthy emotions, and gut-microbiota equilibrium all play a role. In short, disrupted gastrointestinal microbiomes play an important role in the development of mental disorders such as insomnia and depression. Thus, identifying new research methods that can be used to help understand the mechanism underlying this relationship is imperative. The following aspects should be addressed. (1) We should investigate the effect of probiotics in maintaining gut-microbiota equilibrium and explore the differences in efficacy between individual probiotics and combinations of multiple probiotics in achieving targeted therapeutic effects. (2) The frequency and timing of stool sample collection is particularly important given recent results demonstrating the circadian rhythms of some gut microbes and their sensitivity to melatonin. Future research should determine how samples collected at different times of day differ to make it possible to establish the best times and frequencies for sample collection. These studies should also clarify the amount of time that elapses from the beginning of a circadian rhythm disturbance to the beginning of changes in the structure and the composition of intestinal microbiota. (3) We should determine to what extent mistimed sleep can cause dysregulation of the intestinal microbiota. It is important to determine the characteristic differences in intestinal microbial structure and function that occur in acute insomnia and chronic insomnia. (4) Lack of sleep, circadian rhythm disorders, bad mood, food energy intake, and the gut microbiota have complex relationships that require further elucidation. With these considerations in mind, identifying any differences between controlled intake and *ad libitum* feeding will be important, particularly when sleep and wake opportunities are altered under shift-working conditions. Simulated shift work studies of humans in controlled environments can be used to begin exploring these relationships. Cohort studies will also play an important role in determining the impact of ongoing shift work in real-world settings, providing the larger sample sizes needed to detect changes when energy intake, and lifestyle are not held constant. (5) We should explore how intestinal microbial changes affect sleep and depression rhythms. Such research will contribute to the rational selection of times for the administration of drugs for the treatment of mental disorders such as insomnia and depression. It will also contribute to maximizing the therapeutic effects of drugs and minimizing drug side effects. (6) A randomized, double-blind clinical study can be used at the individual level to reduce the interference and confounding bias introduced by human subjective factors on test results. Such studies will allow the evaluation of therapeutic effects on insomnia based on the regulation of certain intestinal microbiota.

## Author Contributions

Each author contributed substantially to the paper. YL conceived the study hypothesis and wrote the main body of the paper. BZ and FF contributed to reviewing the literature and supervised the writing of the manuscript. YH performed the methodological search on the research topic and helped write the draft manuscript. All authors approved the final version of the manuscript.

### Conflict of Interest Statement

The authors declare that the research was conducted in the absence of any commercial or financial relationships that could be construed as a potential conflict of interest.
